# The effect of neighborhood social cohesion on life satisfaction in type 2 diabetes mellitus patients: the chain mediating role of depressive symptoms and sleep quality

**DOI:** 10.3389/fpubh.2023.1257268

**Published:** 2023-12-15

**Authors:** Xueping Ma, Wei Bai, Fan Yu, Fan Yang, Jiaxin Yin, Huilian Shi, Yang Niu, Liqun Wang

**Affiliations:** ^1^Heart Centre & Department of Cardiovascular Diseases, General Hospital of Ningxia Medical University, Yinchuan, China; ^2^Department of Pulmonary Disease, Zhongwei Hospital of Traditional Chinese Medicine, Zhongwei, China; ^3^School of Traditional Chinese Medicine, Ningxia Medical University, Yinchuan, China; ^4^Key Laboratory of the Ningxia Ethnomedicine Modernization, Ministry of Education, Ningxia Medical University, Yinchuan, China; ^5^Department of Epidemiology and Health Statistics, School of Public Health, Ningxia Medical University, Yinchuan, China; ^6^Key Laboratory of Environmental Factors and Chronic Disease Control, Ningxia Medical University, Yinchuan, China

**Keywords:** neighborhood social cohesion, depressive symptoms, sleep quality, life satisfaction, mediating effect, T2DM

## Abstract

**Objective:**

Although most studies have shown that neighborhood social cohesion (NSC) is associated with life satisfaction among patients with type 2 diabetes mellitus (T2DM), it remains unclear how NSC is linked to life satisfaction. The present study aims to examine the potential mediation effect of depressive symptoms and sleep quality on the relationship between NSC and life satisfaction among Chinese individuals with T2DM.

**Methods:**

A cross-sectional survey was conducted from August 2019 to November 2020 involving 1747 T2DM patients. The main information was obtained using the Center for Epidemiological Survey Depression Scale (CES-D), self-report sleep quality and life satisfaction questionnaire and NSC scales. Mediation analyses were performed using the PROCESS macro in SPSS.

**Results:**

The results manifested that the NSC was positively associated with sleep quality (r = 0.219) and life satisfaction (r = 0.214), while negatively correlated with depressive symptoms (r = −0.232). Depressive symptoms were found to be negatively associated with life satisfaction (r = −0.263). NSC influenced life satisfaction through three mediating pathways: (a) depressive symptoms (effect = 0.0081); (b) depressive symptoms and sleep quality (effect = 0.0019); and (c) sleep quality (effect = 0.0015). The total mediating effect accounted for 28.1% of the overall effect.

**Conclusion:**

Our findings support the hypothesis that depressive symptoms and sleep quality mediated the relationship between NSC and life satisfaction in patients with T2DM. It is important to encourage T2DM patients to participate in social interactions and enhance their level of NSC. Additionally, efforts should be made to actively reduce depressive symptoms and improve sleep quality, so as to improve their life satisfaction.

## 1 Introduction

As one of the most serious and common chronic diseases of the present day, diabetes causes life-threatening, severe complications and reduces life quality (1). Recent estimates from 2021 indicate that diabetes affects approximately 10% of individuals aged 20 to 79 globally, with a projected increase to 12.2% by 2045, amounting to a staggering 783.2 million cases (2). Diabetes prevalence in China has increased every year, reaching 12.4% in 2018 (3). Type 2 diabetes mellitus (T2DM) is the most prevalent form of diabetes and accounts for the majority of diabetes cases worldwide. It imposes a higher burden on healthcare systems due to its chronic nature and associated complications (4) and is a tremendous threat to human life satisfaction.

Life satisfaction is the component of subjective well-being that is based on cognitive-judgmental assessment. It can be defined as a cognitive and global evaluation of the quality of their life as a whole (5), and the quality of life is related to physical and mental health of T2DM patients (6). There exists evidence that life satisfaction may offer health benefits (7); Many studies indicate that life satisfaction may be linked to health status (8) or chronic disease (9).

Life satisfaction was influenced by a range of factors, but previous studies mainly focused on the negative effects, such as anxiety (10), depression (11), and environmental degradation (12). There was no consideration given to the benefits caused by positive characteristics, such as neighborhood social cohesion (NSC) that enhance life satisfaction. As a type of social capital, NSC refers to the degree of perceived connection between neighbors, as well as social bonds, shared values, and the norms of residents in a neighborhood (13). As a social determinant of health, the role of NSC has attained considerable attention in the field of public health (9). Studies have shown that.

NSC in others was associated with a wide range of health and well-being outcomes, including self-rated health (14), mental health (15), quality of life (16), subjective well-being (17), etc. Despite increasing attention that addresses the significance of NSC on health, very few studies have examined life satisfaction among T2DM patients. Moreover, the mechanisms that explain the association between NSC and life satisfaction are still unclear.

Theoretically, NSC may be associated with life satisfaction among T2DM patients through the suppression of depressive symptoms or the improvement of sleep quality. This pathway may be particularly critical to investigate among patients with type 2 diabetes given associations between this chronic health condition and poor life satisfaction. A cohort study involving 15,438 participants from Central and Eastern Europe showed that increased levels of depressive symptoms were found to be linked to reduced social cohesion during a 3-year follow-up period among older individuals. (18). Our previous study indicated a strong correlation between NSC and depressive symptoms in T2DM patients (16). Worth noting, depressive symptoms were highly associated with life satisfaction in a nationwide sample of healthy Finnish adults (*N* = 9,679) (19), but not reported in diabetes patients. Additionally, sleep quality, being an essential human need, plays a vital role in maintaining good health. A previous study included 1756 older Chinese adults found that poor sleep quality was inversely related to life satisfaction (20). However, gaps still exist in the knowledge of sleep quality and life satisfaction among T2DM patients.

Given this backdrop, this study aims to explore the influence mechanism of NSC on T2DM patients’ life satisfaction and construct a chain mediation model of NSC on T2DM patients’ life satisfaction with depressive symptoms and sleep quality as mediating variables. The current study aims to: (i) examine whether NSC, depressive symptoms, and sleep quality could significantly predict T2DM patients’ life satisfaction; and (ii) explore the mediating effects of depressive symptoms and sleep quality on the relationship between NSC and life satisfaction among T2DM patients.

## 2 Materials and methods

### 2.1 Study design and participants

A cross-sectional study was conducted based on 1,747 T2DM inpatients enrolled from 10 public hospitals in Ningxia province from August 2019 to November 2020. The following are the inclusion criteria: (1) ≥18 years of age; (2) living at their residence for more than six months. Meanwhile, participants were excluded if they (1) had a severe mental disorder or a severe illness (2) had a malignant tumor (3) with sleep disorders and taking hypnotic medications, or some particular occupation need to go to bed late. The Yinchuan Hospital of Traditional Chinese Medicine Institutional Review Board approved the study. All the participants provided a written consent form prior to the survey.

### 2.2 Life satisfaction

In light of the suffering of patients, we used a single question rather than a scale to gage life satisfaction. The following question was asked, “How satisfied are you with your current life?” The possible responses are “very dissatisfied,” “somewhat dissatisfied,” “neither satisfied nor dissatisfied,” “somewhat satisfied,” and “very satisfied,” scored from 1 to 5. The validity and reliability of this question have been demonstrated in many countries (21, 22).

### 2.3 Neighborhood social cohesion

Neighborhood social cohesion (NSC) was assessed using an adapted scale of four items developed by Mujahid and his colleagues (23), to measure participants’ evaluations (from 1 = Strongly disagree to 5 = Strongly agree) with regard to their neighbors: (1) People around here are willing to help their neighbors, (2) People in my neighborhood generally get along with each other, (3) People in my neighborhood can be trusted, and (4) People in my neighborhood share the same values. The scale has been used among the general Chinese population with good internal consistency (Cronbach’s α: 0.81) (24). The Cronbach’s alpha in this sample was 0.95.

### 2.4 Depressive symptoms

A 20-item Center for Epidemiological Studies Depression Scale (CES-D-20) (25) was used to assess depressive symptoms over the past week (25). Scores are based on 0 to 3 for each item, a score of 0 indicates that the item occurs rarely or never less than one day, and 3 indicates that it occurs all the time, five to seven days (26). The total score ranges from 0 to 60, a higher score indicates a higher level of depression. The scale has been used to measure depressive symptoms among different T2DM patients and shown to be reliable (26, 27). The Cronbach’s alpha in this sample was 0.88.

### 2.5 Sleep quality

We set the question “How do you think of your sleep quality?” to determine the sleep quality, with answers poor, general, and good, and scored 1, 2, 3, respectively. The question has been used to measure sleep quality and shown to be reliable (28).

### 2.6 Covariates

Covariates consist of the demographic characteristics and health-related behaviors or conditions. The demographic characteristics included age (in years), gender (male vs. female), residence (urban vs. rural), educational level (illiterate, primary, junior and senior, college degree and above), marital status (unmarried, married and widowed or divorced), economic situation (family average individual income per month and was divided into two groups: <2,000 yuan (RMB) and ≥ 2,000 yuan (RMB)), and residence (rural vs. urban) were collected via a self-administrated questionnaire. The health-related behaviors or condition included smoking (defined as six months or more of smoking at least one cigarette a day), alcohol use (During the past year, drank at least one glass of alcohol, equivalent to half a bottle of beer, 125 milliliters of grape wine, fruit wine, or 40 milliliters of white wine), physical activity (Do you engage in at least half an hour of physical activity (including normal daily activities) during work and/or leisure time every day?), body mass index (BMI = weight (kg)/height (m)^2^), other chronic diseases (yes vs. no), T2DM complications (yes vs. no), take medicine (yes vs. no). disease duration (years), and sleep duration (hours).

### 2.7 Statistical analyses

The software SPSS 25.0 (IBM Corp., Armonk, NY, United States) was used to perform the statistical analyses. Frequencies and percentages [*n* (%)] were used to describe the demographic characteristics of the participants, while the continuous data conforming to the normal distribution was represented by ( $\bar{\chi }$ ± s). The coefficient of internal consistency and the internal consistency reliability were measured using Chronbach’s alpha values method. A correlation matrix was constructed using partial correlation analysis after controlling the covariances. The linear regression model was used to examine the influencing factors of life satisfaction. Variables related to the independent (NSC) and dependent (life satisfaction) variables were adjusted as confounders. In all tests, two-sidedness was used and 0.05 was considered significant. The mediating effect was evaluated using PROCESS bootstrap methods (29). Major test: the direct effect relationship between NSC and life satisfaction; mediating effect of depressive symptoms and sleep quality; the chain mediating effect of NSC and life satisfaction. Mediation analysis was conducted using life satisfaction as a continuous variable, and using PROCESS macro Model 6, with 5,000 bias-corrected bootstrap resamples; bias-corrected percentile bootstrap confidence interval (CI) was used to evaluate the effect size, a 95% CI that did not include zero indicates statistical significance (30). The proportion mediated was calculated by dividing the indirect effect by the total effect.

## 3 Results

### 3.1 Demographic characteristics

A description of the demographic characteristics of the participants were displayed in Table 1. In total, the average age of the participants was 58.6 years old, with more than half (54.8%) of the participants being males, and most of them obtained from urban area (62.7%), and were married (93.3%). The mean social cohesion score was 15.2 ± 3.1 points, and the depressive symptoms score was 11.1 ± 7.3 points. Approximately 80% of participants with higher life satisfaction.

**Table 1:** Demographic characteristics of T2DM patients (*n* = 1,747).

Variables	*N* (%)/( $\bar{\chi }$ ± s)
Age, years old, ( $\bar{\chi }$ ± s)	58.6 ± 12.1
Gender, male, n (%)	958(54.8)
Marital status, married, *n* (%)	1,630(93.3)
Educational attainment, *n* (%)	
Illiterate	348(19.9)
Primary	392(22.4)
Junior or senior	616(35.3)
bachelor and above	391(22.4)
Economic level, yuan, <2000, *n* (%)	680(38.9)
Residence, urban, *n* (%)	1,095(62.7)
Smoking, yes, *n* (%)	393(22.5)
Alcohol use, yes, *n* (%)	365(20.9)
Physical activity, yes, *n* (%)	1,169(66.9)
BMI, kg/m^2^, ( $\bar{\chi }$ ± s)	24.98 ± 5.97
Other chronic diseases, yes, *n* (%)	1,149(65.8)
T2DM complications, yes, *n* (%)	1,048(60.0)
Take medicine, yes, *n* (%)	1,457(83.4)
Disease duration, year, ( $\bar{\chi }$ ± s)	8.3 ± 7.5
Sleep duration, hour, ( $\bar{\chi }$ ± s)	8.3 ± 1.1
Life satisfaction, *n* (%)	
Very dissatisfied	3(0.2)
somewhat dissatisfied	40(2.3)
neither satisfied nor dissatisfied	298(17.1)
somewhat satisfied	1,252(71.7)
Very satisfied	154(8.8)
Social cohesion, points, ( $\bar{\chi }$ ± s)	15.2 ± 3.1
Depressive symptoms, points, ( $\bar{\chi }$ ± s)	11.1 ± 7.3
Sleep quality, *n* (%)	
Poor	393(22.5)
General	783(44.8)
Good	571(32.7)

SD = standard deviation, BMI = body mass index, T2DM = type 2 diabetes mellitus.

### 3.2 Correlation analysis

As shown in Table 2, the NSC was positively associated with sleep quality (*r* = 0.219, *p* < 0.001) and life satisfaction (*r* = 0.214, *p* < 0.001), while negatively depressive symptoms (*r* = −0.232, *p* < 0.001). And depressive symptoms were negatively associated with life satisfaction (*r* = −0.263, *p* < 0.001).

**Table 2:** Correlation matrix (*n* = 1,747).^a^

Variables	Mean	SD	Life satisfaction	Social cohesion	Depressive symptoms	Sleep quality
Life satisfaction	3.87	0.59	1	-	-	-
Social cohesion	15.18	3.12	0.214*	1	-	-
Depressive symptoms	11.07	7.31	−0.263*	−0.232*	1	-
Sleep quality	2.10	0.74	0.219*	0.123*	−0.310*	1

*p < 0.001, SD = standard deviation.

a After controlling for age, gender, marital status, educational level, residence, economic status, smoking, alcohol use, physical activity, BMI, other chronic diseases, T2DM complications, take medicine, disease duration, sleep duration.

### 3.3 Linear regression analysis

As shown in Table 3, we found age (β = 0.04, *p* = 0.012), educational level (β = 0.05, *p* = 0.016), smoking (β = 0.12, *p* = 0.002), physical activity (β = 0.09, *p* = 0.002), NSC (β = 0.03, *p* < 0.001) and sleep quality (β = 0.12, *p* < 0.001) were statistically and positively related to life satisfaction. The individuals who were male (β = −0.12, *p* < 0.001), and with depressive symptoms (β = −0.01, p < 0.001) were negatively associated with life satisfaction.

**Table 3:** Linear regression model of predictors of life satisfaction among T2DM patients (*n* = 1,747).

Variables	Crude β (95% CI)	*p*	Adjusted β (95% CI)	*p*
Age	−0.01(−0.03,0.02)	0.624	0.04(0.01,0.07)	0.012
Gender(reference = female)	−0.01(−0.07,0.04)	0.670	−0.12(−0.19,-0.06)	<0.001
Marital status(reference = unmarried)	0.15(0.04,0.26)	0.008	0.07(−0.03,0.18)	0.177
Educational level(reference = illiterate)	0.04(0.01,0.06)	0.006	0.05(0.01,0.08)	0.016
Economic condition (reference = less than 2000 yuan)	0.02(0.01,0.05)	0.030	0.01(−0.02,0.03)	0.748
Residence(reference = rural)	0.06(0.01,0.12)	0.036	0.02(−0.05,0.08)	0.590
Smoking(reference = no)	0.07(0.01,0.14)	0.038	0.12(0.04,0.19)	0.002
Alcohol use(reference = no)	0.06(−0.01,0.13)	0.096	0.01(−0.06,0.09)	0.737
Physical activity(reference = no)	0.13(0.07,0.19)	<0.001	0.09(0.03,0.14)	0.002
BMI	−0.01(−0.05,0.03)	0.618	−0.01(−0.05,0.02)	0.570
Other chronic diseases(reference = no)	−0.03(−0.09,0.03)	0.276	−0.02(−0.07,0.04)	0.588
T2DM complications(reference = no)	−0.03(−0.09,0.02)	0.274	−0.02(−0.07,0.04)	0.560
Take medicine(reference = no)	0.05(−0.02,0.13)	0.147	0.04(−0.03,0.11)	0.248
Disease duration	−0.001(−0.005,0.003)	0.622	−0.002(−0.006,0.002)	0.278
Sleep duration	−0.03(−0.05,-0.01)	0.013	−0.02(−0.05,0.00)	0.053
Social cohesion	0.04(0.03,0.05)	<0.001	0.03(0.02,0.04)	<0.001
Depressive symptoms	−0.02(−0.03,−0.01)	<0.001	-0.01(−0.02,-0.01)	<0.001
Sleep quality(reference = poor)	0.16(0.13,0.20)	<0.001	0.12(0.08,0.15)	<0.001

95%CI: 95% confident interval.

### 3.4 Mediation analysis

**Figure 1:**
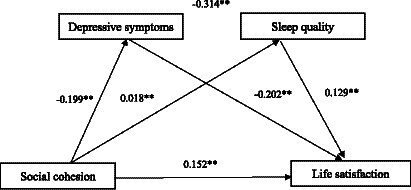
Mediating effect analysis of depressive symptoms / sleep quality between social cohesion and life satisfaction; * *p* < 0.01; ** *p* < 0.001.

Based on the mediation model, both the direct effect (95%CI:0.0208–0.0383) and the total indirect effect were significant (95%CI:0.0083–0.0150). The indirect effects of social cohesion on life satisfaction via depressive symptoms, sleep quality, and both were 0.0081 (95% CI: 0.0054–0.0115), 0.0015 (95% CI, 0.0002–0.0032), and 0.0019 (0.0012, 0.0028), respectively. The mediation effect accounted for 28.1% (0.0115/0.0410) of the total effect (see Table 4). The final path model was shown in Figure 1.

**Table 4:** The mediation effect of depressive symptoms and sleep quality on the relationship between social cohesion and life satisfaction (*n* = 1,747).^a^

Effect types	*Effect*	*SE*	*p* value	Bias-Corrected *95%CI*	
				Lower	Upper
Total effect	0.0410	0.0045	<0.001	0.0322	0.0499
Direct effects	0.0296	0.0045	<0.001	0.0208	0.0383
Indirect effects (depressive symptoms)	0.0081	0.0015	<0.001	0.0054	0.0115
Indirect effects (sleep quality)	0.0015	0.0008	0.001	0.0002	0.0032
Indirect effects (depressive symptoms and sleep quality)	0.0019	0.0004	0.001	0.0012	0.0028
Total indirect effects	0.0115	0.0017	-	0.0083	0.0150

SE: standard error; 95%CI: 95% confident interval.

a All the analysis under controlling for age, gender, marital status, educational level, residence, economic status, smoking, alcohol use, physical activity, BMI, other chronic diseases, T2DM complications, take medicine, disease duration, sleep duration.

## 4 Discussion

In the present investigation, a cross-sectional study was undertaken to examine the correlation between NSC, depressive symptoms, sleep quality, and life satisfaction in individuals with T2DM, as well as to analyze the mediating role of depressive symptoms and sleep quality in this relationship. As postulated, the findings revealed the following: (1) NSC and sleep quality exhibited a positive association with life satisfaction, whereas depressive symptoms displayed an inverse relationship with life satisfaction; (2) Depressive symptoms and sleep quality played a significant mediating role in the association between NSC and life satisfaction, accounting for 28.1% of the total effect.

This study revealed that depressive symptoms and sleep quality play a chain intermediary role (β =0.0019, 95% CI = [0.0012, 0.0028], *p* = 0.001) in the relationship between NSC and life satisfaction, suggesting that the mediating effect of depressive symptoms and sleep quality was established. In the analysis model, NSC can not only directly associated with the T2DM patients’ life satisfaction, but also indirectly, by depressive symptoms and sleep quality, and both of them, associated with T2DM patients’ life satisfaction. Based on the findings, we found that NSC could elevate T2DM patients’ life satisfaction, as well as alleviate depression mood and improve their sleep quality by means of enhancing NSC so as to obtain greater sleep quality and improve life satisfaction. Wakefield et al. observed a positive relationship between depression and sleep quality in their study (31). Besides, another study reported a statistically significant positive correlation between depression and sleep quality (32). The underlying mechanism for these findings could be linked to the association between NSC and a sense of belonging, which may enhance T2DM patients’ capacity to resist depressive symptoms (33, 34). This, in turn, may contribute to creating a comfortable sleep environment and achieving good sleep quality.

Our findings indicate that NSC is a significant predictor of life satisfaction among T2DM patients (β = 0.03, *p* < 0.001), aligning with previous research suggesting that enhancing well-being and life satisfaction can be achieved through NSC or social capital (35, 36). Moreover, a previous study has demonstrated a positive correlation between high levels of NSC and improved quality of life in T2DM patients (16). Additionally, our study reveals a negative association between depressive symptoms and life satisfaction (β = −0.01, p < 0.001). A previous study reported that lower depressive symptoms were associated with higher life satisfaction levels among patients with multiple system atrophy (37). Furthermore, our study revealed a significant correlation between sleep quality and life satisfaction, aligning with prior research that suggests a link between sleep quality and life satisfaction in older adults (38). In summary, to effectively address the issue of low life satisfaction in patients with Type 2 Diabetes Mellitus (T2DM), it is imperative to not only enhance the sleep quality of T2DM patients but also mitigate their depressive symptoms in order to elevate their overall life satisfaction.

### 4.1 Limitations

A strength of this study was the application of mediation analysis that provided primary evidence for understanding the mechanisms of how NSC is associated with life satisfaction in a large sample of T2DM patients in northwest China, further research may also be spurred as a result. However, several limitations existed in the present study. First, this was a cross-sectional design that precluded the derivation of cause-effect relationships between variables. Second, we used self-reported data of sleep quality and it may involve information bias even though it is commonly used in the epidemiological study due to the feasibility consideration. Third, when promoting and applying the results to other countries should be cautious because this study was carried out with a Chinese sample.

## 5 Conclusion

The findings of the study indicate that NSC has a positive effect on life satisfaction among individuals with T2DM, and depressive symptoms and sleep quality played a chain mediating role between NSC and life satisfaction. The mediation effect suggests that those two variables have important practical significance in improving T2DM patients’ life satisfaction. Hence, it is necessary to guide our T2DM patients to engage in more social communication (chat with others or exchange information with other patients) and obtain high level of NSC, and to actively reduce depressive symptoms and improve their sleep quality (consult a psychologist or do some exercises), so as to improve their life satisfaction. The study findings will provide a reference for clinical doctors and community health service staff to improve the social participation and life satisfaction of T2DM patients in the future.

## Data availability statement

The raw data supporting the conclusions of this article will be made available by the authors, without undue reservation.

## Ethics statement

The studies involving humans were approved by The Yinchuan Hospital of Traditional Chinese Medicine Institutional Review Board approved the study. The studies were conducted in accordance with the local legislation and institutional requirements. The participants provided their written informed consent to participate in this study.

## Author contributions

XM: Conceptualization, Data curation, Investigation, Writing – original draft. WB: Data curation, Investigation, Writing – original draft. FYu: Data curation, Investigation, Writing – original draft. FYa: Data curation, Investigation, Writing – original draft. JY: Data curation, Investigation, Writing – original draft. HS: Data curation, Investigation, Writing – original draft. YN: Conceptualization, Project administration, Writing – review & editing. LW: Conceptualization, Methodology, Project administration, Writing – original draft, Writing – review & editing.
